# Multifunctional role of zinc in human health: an update

**DOI:** 10.17179/excli2023-6335

**Published:** 2023-08-04

**Authors:** Despoina P. Kiouri, Evi Tsoupra, Massimiliano Peana, Spyros P. Perlepes, Maria E. Stefanidou, Christos T. Chasapis

**Affiliations:** 1 Institute of Chemical Biology, National Hellenic Research Foundation, 11635 Athens, Greece; 2 Department of Chemistry, Laboratory of Organic Chemistry, National Kapodistrian University of Athens, 15772 Athens, Greece; 3 Department of Chemistry, University of Patras, 26504 Patras, Greece; 4 Department of Chemical, Physical, Mathematical and Natural Sciences, University of Sassari, 07100 Sassari, Italy; 5 Department of Forensic Medicine and Toxicology, School of Medicine, National and Kapodistrian University of Athens, 11527 Athens, Greece

**Keywords:** zinc, cancer, gut microbiome, severe acute respiratory syndrome coronavirus 2, Alzheimer's disease, aging, immune system

## Abstract

Zinc is a multipurpose trace element for the human body, as it plays a crucial part in various physiological processes, such as cell growth and development, metabolism, cognitive, reproductive, and immune system function. Its significance in human health is widely acknowledged, and this has led the scientific community towards more research that aims to uncover all of its beneficial properties, especially when compared to other essential metal ions. One notable area where zinc has shown beneficial effects is in the prevention and treatment of various diseases, including cancer. This review aims to explain the involvement of zinc in specific health conditions such as cancer, coronavirus disease 2019 (COVID-19) and neurological disorders like Alzheimer's disease, as well as its impact on the gut microbiome.

## Abbreviations

Acetyl-CoA Acetyl coenzyme A

Ach Acetylcholine

AChE Acetylcholinesterase

AD Alzheimer's disease

ADHD Attention deficit hyperactivity disorder

APP Amyloid precursor protein

ASD Autism spectrum disorder

ATP Adenosine 5′-triphosphate

ChAT Choline acetyltransferase

COVID-19 Coronavirus Disease 2019

CP Calprotectin

CQ Clioquinol

Cu Copper 

Cu/Zn-SOD Copper/Zinc Superoxide Dismutase

CZNPs Chitosan-assembled zinc oxide nanoparticles

DDR DNA damage response

DNMTs DNA methyltransferases

EMT Epithelial Mesenchymal Transition

ERK Extracellular Signal Regulated Kinases

GFAP Glial fibrillary acidic protein

GM Gut Microbiome

H_2_O_2_ Hydrogen peroxide 

HATs Histone acetyltransferases

HCoVs human-infecting CoVs

HCV Hepatitis C virus

HDACs Histone deacetylases

HIV Human Immunodeficiency virus

ICU Intensive care unit

IL Interleukin

LPS Lipopolysaccharide

m-aconitase Mitochondrial aconitase

MAPT Microtubule-associated protein tau

MCI Mild cognitive impairment

MERS-CoV Middle East respiratory syndrome coronavirus

MTs Metallothioneins

mZn Zinc 'mobile' form

NDs Neurodegenerative diseases

NFTs Neurofibrillary tangles

NSAIDs Non-steroid anti-inflammatory drugs

O^2^ Superoxide anion free radical

PDT Photodynamic therapy

ROS Reactive oxygen species

SARS-CoV-2 Severe acute respiratory syndrome coronavirus

SMON Subacute myelo-optico-neuropathy

Ybt Yersiniabactin

ZIPs Zn-Importing Proteins

ZIPs Zrt-/Irt-like proteins

Zn Zinc 

ZnC Zinc carnosine

ZnO NPs Zinc oxide nanoparticle

ZnTs Zn Transporters 

## Introduction

In Biochemistry Zn^2+^ is classified as a trace element, the second most abundant in humans after iron. It has regulatory, structural, and catalytic roles and it is present as a component in more than 2500 proteins, including enzymes and transcription factors (Andreini et al., 2006[2]; Kambe et al., 2015[36]). Approximately 10 % of the human proteome is associated with Zn^2+^ ions, which play a significant role for regulation of gene expression, metabolism of DNA, chromatin structure, cell proliferation, apoptosis, immunity, cognition and defenses towards oxidants (Andreini et al., 2006[2]; Costa et al., 2023[15]). In addition, it regulates intracellular signal transduction and is critical for efficient synaptic transmission in the central nervous system (Portbury and Adlard, 2017[71]). Zinc is an essential mineral for the proper function of the human body (Zoroddu et al., 2019[116]). For example, zinc can improve chondrocyte and osteoblast function while reducing osteoclast activity, indicating a role for the metal in bone homeostasis and regeneration (O'Connor et al., 2020[67]). It is involved in numerous physiological processes as summarized in Table 1(Tab. 1).

It is important to get an adequate amount of zinc through diet or, if necessary, through supplements, to ensure proper functioning of the body and to prevent zinc deficiencies, which can cause a variety of health problems (Zoroddu et al., 2019[116]). However, it's important to note that too much zinc can be harmful, so it's important to follow the recommended guidelines for daily zinc intake. Moreover, zinc plays an important role in the prevention of various diseases. Since one of the main roles of zinc is the support of the immune system, a zinc deficiency state can make the body more susceptible to infection and disease (Chasapis et al., 2020[14]; Maares and Haase, 2016[54]). A sufficient zinc intake can help boost immunity and prevent respiratory infections of all kinds, as well as other common illnesses (Skalny et al., 2020[87]; Stefanidou et al., 2006[92]). Zinc has anti-inflammatory and antimicrobial properties, which can help prevent or reduce the occurrence of acne, dermatitis and other skin-related conditions (Zou et al., 2023[117]). Additionally, zinc is involved in the synthesis of collagen, which is essential for maintaining healthy skin (Molenda and Kolmas, 2023[63]). An increasing body of research suggests that hyperglycemia and zinc metabolism are linked, and also that zinc supplementation could contribute to the improvement of glycemic control in diabetic patients (Wang et al., 2019[101]). Because Zn is fundamental for the regulation of both biochemical and metabolic processes, its deficiency is thought to be an important factor of obesity development (Rios-Lugo et al., 2020[79]). Several studies have shown that adequate zinc intake can reduce the risk of cardiovascular diseases, such as atherosclerosis and coronary heart disease (Knez and Glibetic, 2021[43]). Zinc plays a key role in keeping blood vessels healthy by improving circulation and reducing inflammation in the arteries (Betrie et al., 2021[6]). In addition, some studies suggest that zinc could be helpful in treating mental disorders, such as depression and anxiety (Anbari-Nogyni et al., 2020[1]; Petrilli et al., 2017[70]). Zinc is also involved in the regulation of serotonin levels, a neurotransmitter associated with mental well-being (Doboszewska et al., 2017[19]). Additionally, zinc is necessary for proper brain function and may help preserve memory and cognitive function over time (Sandusky-Beltran et al., 2017[83]). Zinc can be helpful in the prevention of certain eye diseases, such as age-related macular degeneration (AMD) and cataracts (Barman and Srinivasan, 2019[4]; Blasiak et al., 2020[7]), and it plays an important role in eye health by protecting the retina and contributing to good vision (Organisciak et al., 2012[68]). There is evidence to suggest a role for zinc in the prevention and treatment of some types of cancer (Chasapis et al., 2012[13]; Hoppe et al., 2021[29]). Some studies suggest that adequate levels of zinc may help prevent the development of certain types of cancer, such as prostate, breast, colon, and esophageal cancer. Zinc may play a role in regulating cell growth, activating enzymes that perform antioxidant functions, and stabilizing DNA (Prasad and Bao, 2019[72]). More specifically, zinc is essential for the functioning of several epigenetic enzymes, including DNA methyltransferases (DNMTs), histone acetyltransferases (HATs), histone deacetylases (HDACs), and histone demethylases (Brito et al., 2020[9]).

Additionally, zinc supplementation may be beneficial during cancer therapy, as some treatments such as chemotherapy can reduce zinc levels in the body (Hoppe et al., 2021[29]). Maintaining adequate zinc levels can help counteract the side effects of therapy, such as weight loss, fatigue, and compromised immune system. Zinc is involved in the functioning of the immune system and in protein synthesis, both of which are relevant factors in the fight against viral infections such as SARS-CoV-2. Additionally, some research suggests that zinc may have antiviral properties and could interfere with virus replication (Read et al., 2019[76]; Wessels et al., 2020[103]). Zinc is also a well-established strategy for the treatment of some pathologies, like Wilson's disease where the available medication for its treatment includes chelators and Zn salts. Also, the relationship between zinc and Alzheimer's is a topic that has been studied in recent years. Some research has suggested that zinc metabolism may be impaired in people with Alzheimer's disease, leading to abnormal zinc levels in the brain (Rivers-Auty et al., 2021[80]). Besides Alzheimer's, the role of zinc and zinc-binding proteins in the aging process has been under investigation. Zinc plays an important part in the health of the gut microbiome, a complex microbial ecosystem plays a vital role in human health, influencing digestion, nutrient absorption, the immune system and even mood (Skalny et al., 2021[86]). 

This review aims to describe the role of zinc in several different pathological states that have been in the limelight, including cancer, COVID-19, neurological disorders such as Alzheimer's disease, as well as its action in the gut microbiome. Importantly, zinc is not a cure, but it can be used as part of an overall prevention strategy to better support health and reduce the risk of these diseases. 

## Zinc and Cancer

One of the metals that are essential to life is zinc, which is the earth's crust 24^th^ most prevalent element and the second most abundant micronutrient in the human body (Gelbard, 2022[23]; Renteria et al., 2022[78]; Zoroddu et al., 2019[116]). It participates in a variety of cellular processes, such as cellular signaling, differentiation and proliferation, homeostasis maintenance, immunological function, oxidative stress and antioxidant responses, apoptosis, and aging, as a structural or catalytic component (i.e., cofactor) in more than 300 enzymes, although according to bioinformatics studies roughly 3000 human proteins are expected to be zinc-bound (Maywald and Rink, 2022[58]; Rusch et al., 2021[82]). Zinc is also vital for the polymeric organization of nucleic acids as well as DNA replication and damage repair (Renteria et al., 2022[78]; Yan et al., 2008[108]). It is widely known that zinc is involved in a variety of diseases, including infectious, metabolic, intestinal, and neurodegenerative. Low nutritional zinc intake and absorption are linked with cancer manifestation, progression and in some cases metastasis. The recommended daily intake ranges between 9-11 mg and the bigger the zinc insufficiency, the more it correlates with disease progression and survival (Gelbard, 2022[23]).

Copper (Cu) zinc (Zn) Superoxide Dismutase (Cu/Zn-SOD) is a metalloenzyme that catalyzes the dismutation of superoxide anion free radical (O_2_^-.^) into molecular oxygen and hydrogen peroxide (H_2_O_2_) (Younus, 2018[110]). Reactive oxygen species (ROS) like O^2-^ due to their highly reactive and unstable nature can cause DNA damage and alter the DNA damage response (DDR), that is especially pertinent in carcinogenesis as multiple elements of this pathway are mutated in cancer (Srinivas et al., 2019[90]). Additionally, according to a recent study, Cu/Zn-SOD regulates the ROS-responsive expression of a variety of genes, particularly oncogenes (Li et al., 2019[48]). Finally, Cu/Zn-SOD could prevent DNA strand breaks caused by superoxide which highlights its significance in cancer therapy (Prasad et al., 2018[73]). As both metals are necessary for the function of the enzyme, it is the serum Cu/Zn ratio, and not their absolute concentrations, that is essential for the assurance of DNA integrity (Zhang et al., 2022[112]). Studies have reported increased Cu/Zn ratio in bladder cancer, but it doesn't seem to further increase as the disease progresses (Mao and Huang, 2013[56]; Mortada et al., 2020[64]). In colorectal and lung cancer patients, the Cu/Zn ratio was elevated in proportion to the stage of the disease (Juloski et al., 2020[35]; Zhang et al., 2022[112]). The serum Cu/Zn ratio is also elevated in thyroid carcinoma patients, and it has been suggested that this proportion could also be used as a biomarker (Kazi Tani et al., 2021[38]). According to a study by Michos et al., noticeable changes in the Cu and Zn plasma levels occur during the menstrual cycle in women as a result of the cyclic fluctuation of plasma levels of the hormone estradiol (Michos et al., 2010[62]). As a result, the altered Cu/Zn ratio has also been observed in several gynecological cancers, including ovarian (Jafari Shobeiri et al., 2011[32]; Lin and Yang, 2021[49]), endometrial (Atakul et al., 2020[3]), and cervical malignancies (Xie et al., 2018[106]; Zhang et al., 2018[113]) that are often hormone related (Michalczyk and Cymbaluk-Płoska, 2020[61]). In general, low serum zinc levels in cancer patients could be the result of zinc elevated zinc consumption by the mutated cancer cells, that is essential of tumor growth and proliferation, as well as membrane integrity (Sravani et al., 2023[89]). Nevertheless, in some cases low levels of serum zinc have been documented. The protein families that preserve zinc homeostasis in the cells are Zrt-/Irt-like proteins (ZIPs), that mediate Zn influx into the cytoplasm, and Zinc transporters (ZnTs), that are responsible for Zn efflux (Wang et al., 2020[100]). In the case of esophageal cancer, Zn plasma levels lower than normal have been observed as a result of ZIP5 overexpression in human esophageal cancer tissue (Jin et al., 2015[33]; Li et al., 2016[46]). Breast cancer patients have high levels of ZIP6 expression, which is connected to the epithelial mesenchymal transition (EMT) and thus is associated with invasion and metastasis (Li et al., 2016[46]). The latter is consistent with the fact that studies have shown that breast cancer cells have 72 % higher intracellular Zn than normal cells and at the same time serum zinc levels are low (Renteria et al., 2022[78]), suggesting that the cellular zinc level could serve as a biomarker for breast cancer (Rusch et al., 2021[82]). In lung cancer however, high ZIP1, ZIP4, ZIP7 and ZIP10 levels have been documented, but at the same time ZnT7 and ZnT9 were also overexpressed, suggesting that the relationship between zinc transporters and serum zinc levels is not linear (To et al., 2020[96]). Reduced zinc in prostate tissues could be utilized as potential biomarker for prostate cancer diagnosis, because the prostate gland is the biggest zinc reservoir in the human body and thus high zinc concentrations are a sign of a healthy prostate (Li et al., 2020[45]). On one hand, high zinc concentrations inhibit the mitochondrial aconitase (m-aconitase) and lead to high concentrations of citrate (Franz et al., 2013[22]). As a result, adenosine 5′-triphosphate (ATP) production is reduced and the transformation of normal prostatic cells to malignant as well as their proliferation is inhibited. Additionally, high Zn facilitates cytochrome c release into the cytosol and can activate Bax-induced apoptotic cell death and modulate the expression of genes that are associated with apoptosis mechanisms (Franz et al., 2013[22]; Han et al., 2009[27]). At the same time, citrate is secreted into the prostatic fluid where it contributes to sperm motility and release (Li et al., 2020[45]). On the other hand, lack of zinc can accelerate the division of both normal and cancerous cells, although through different mechanisms (Li et al., 2020[45]; To et al., 2020[96]).

It has been demonstrated that zinc nanoparticles and zinc in various combinations are effective anti-cancer and tumor suppressors, both *in vitro *and* in vivo*, with no negative impact on normal cells (Gelbard, 2022[23]; Islam et al., 2022[30]). In the case of cervical cancer cells, zinc oxide nanoparticles (ZnO NPs) and chitosan-assembled zinc oxide nanoparticles (CZNPs) have demonstrated considerable cytotoxicity in a concentration-dependent way (Wu and Zhang, 2018[105]). As ZnO NPs are also easily dissolved at low pH values, they make exceptional pH-sensitive nanocarriers for tumor-targeted drug delivery and release. Besides that, ZnO NPs have the potential to target various cancer cells types, including cancer stem cells and macrophages by hydroxyl radicals, superoxide anions and perhydroxyl radicals production form the surface of the nanoparticle and at the same time by reducing cancer growth, sensitizing drug-resistant tumors, reducing the incidence of cancer recurrence and metastasis and reviving immunosurveillance (Wang et al., 2017[98]). Additionally, zinc has demonstrated remarkable results when evaluated as a photosensitizer in photodynamic therapy (PDT), a technique that is used in anti-tumor treatment (Gunaydin et al., 2021[25]). Finally, zinc can enhance the therapeutic effect of drugs used in cancer therapy, like Paclitaxel and Disulfiram (Gelbard, 2022[23]).

## Zinc and Gut Microbiome

**Figure 1:**
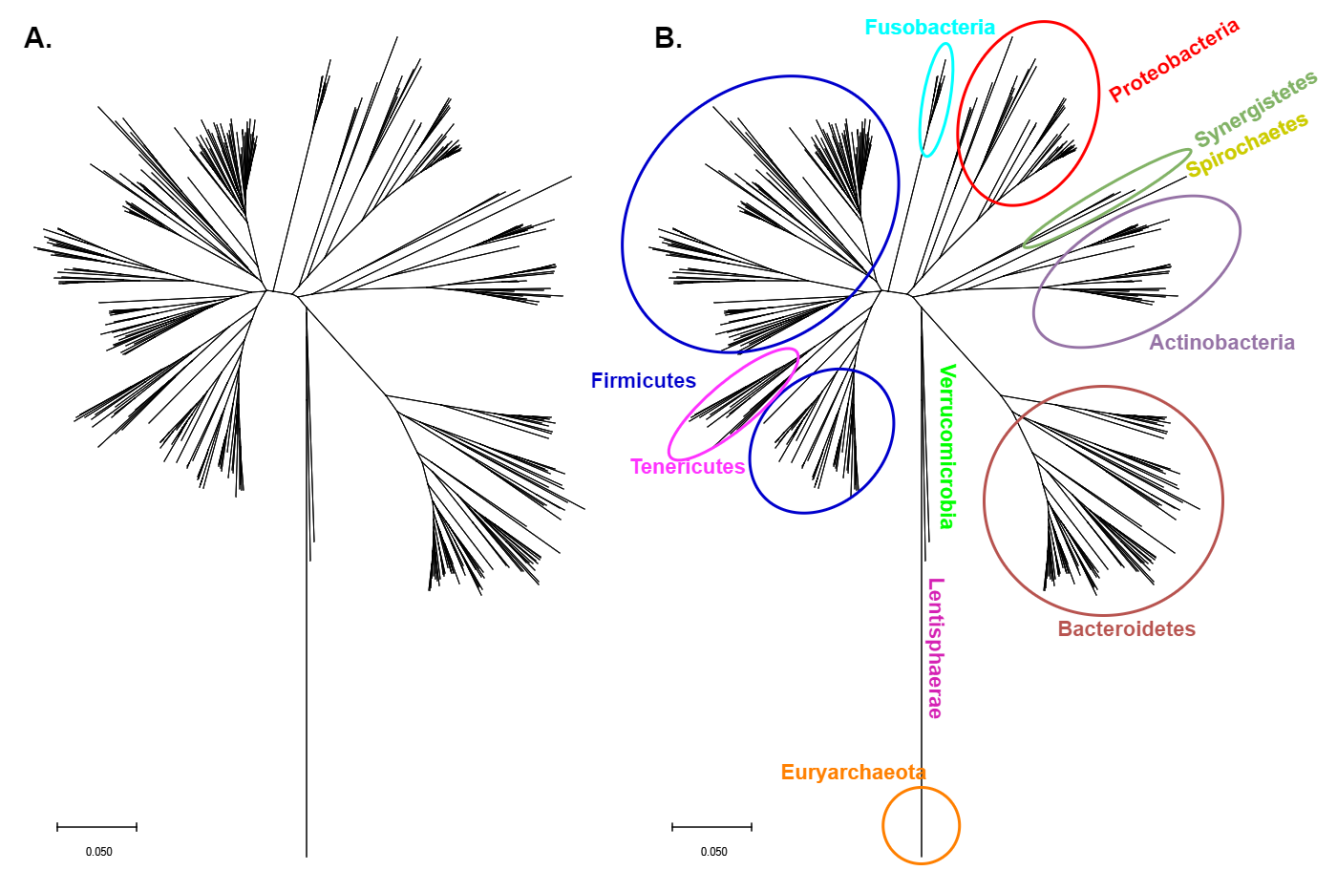
Phylogenetic tree of the 16S ribosomal RNA of the bacteria in the gut microbiome. The multiple sequence alignment (MUSCLE algorithm) and the Phylogenetic tree generation (Neighbor-joining algorithm) were performed in MEGA (version 11) (Tamura et al., 2021).

Human health and disease are significantly influenced by the human microbiome, that is made up of numerous microorganisms that live on the epithelial barrier of the host, including bacteria, fungi, archaea, protozoa, as well as viruses (Li et al., 2019[47]; Stefanidou et al., 2011[91]). The majority of the bacteria that are part of the gut's microbiome (GM) form symbiotic relationships with the host and are essential for the establishment and balance of the intestinal innate and adaptive immunity and, by extension, the maintenance of homeostasis (Malard et al., 2021[55]). The phylogenetic tree of the 16S ribosomal RNA of the bacteria in the gut microbiome can be seen in Figure 1(Fig. 1) (Reference in Figure 1: Tamura et al., 2021[94]). Most of the research has focused on the impact of macronutrients in GM composition, although there have been some studies that have investigated the influence of micronutrients, like zinc, on the microbiota populations of the gut (Davis et al., 2022[17]). In the seventies it was discovered that the GM exploits around 20 % of the dietary zinc (Smith et al., 1972[88]), and changes in dietary zinc intake directly impact the host's GM, as it was described by Reed et al. on a *Gallus gallus* model (Reed et al., 2015[77]). Zinc deficiency has been associated with a decrease in *Verrucomicrobia* and *Proteobacteria* and an increase in *Firmicutes*, *Actinobacteria* and *Bacteroidetes* in mice (Lopez and Skaar, 2018[51]; Mayneris-Perxachs et al., 2016[57]). Low zinc levels and consequently gut microbiota alterations have not only been connected with decreased levels of tight junction markers and a rise in liver lipopolysaccharide (LPS) concentrations, but also with high levels of interleukin (IL) 6 and glial fibrillary acidic protein (GFAP) in the brain (Skalny et al., 2021[86]). The latter indicate that changes in gut microbiota populations directly affect the gut wall permeability and inflammation and are indicative of neuroinflammation (Skalny et al., 2021[86]). It has been proposed that the microbiome-induced neuroinflammation could have an impact on the brain modifications that are observed in autism spectrum disorder (ASD), attention deficit hyperactivity disorder (ADHD) and depression (Sauer et al., 2021[85]; Skalny et al., 2021[86]). Moreover, in ADHD and depression patients, lower bacterial richness has been noted (Kelly et al., 2016[39]; Zhou et al., 2021[115]).

However, one micronutrient alone may not be sufficient for the development of GM alterations and that is the rationale behind studies that consider interacting factors along with zinc deficiency (or low zinc intake), such as pregnancy and aging (Davis et al., 2022[17]). Larger populations of *Actinobacteria*, *Proteobacreria* and *Tenericutes* as well a decrease of *Firimicutes* have been observed in the GMs of autistic individuals, and these changes in particular are thought to be influenced by lower zinc levels in the maternal intestinal flora (Sauer et al., 2021[85]). In older mice, *Lachnospiraceae*, *Acetitafactor*, *Lactobacillus* and *Ruminococcaceae* respond differently to zinc than in younger mice and therefore consist of biomarkers for inflammation and zinc status (Davis et al., 2022[17]). Consequently, GM composition also affects zinc's bioavailability and absorption from food intake (Craig et al., 2021[16]).

In the gut microbiota of healthy individuals, most of the bacteria are part of the *Bacteroides* and *Firmicutes* (Behnsen et al., 2021[5]). In cases of inflamed gut, the microenvironment is altered due to the oxidative conditions and the composition of the gut microbiota. Those conditions are ideal for the growth of facultative anaerobes, like *Enterobacteriaceae* that are mainly linked to inflammatory diseases and obesity (Wang et al., 2020[97]). The pathogenic bacteria also benefit from the elevation of certain nutrients and some of the host's metabolism by-products that further support their growth at the expense of the obligate anaerobes of the GM (Guo et al., 2020[26]). Additionally, the pathogenic bacteria gain functions as a result of inflammation-facilitated metabolic reprogramming, like changes in transcriptional regulation and horizontal gene transfer (Guo et al., 2020[26]), that are crucial for their adaptation in the conditions of the inflamed gut. One of the main mechanisms that these bacteria use for survival is metal nutrient scavenging. During homeostasis, the concentration of metal ions is strictly regulated by the host cells themselves and the GM (Behnsen et al., 2021[5]). Nevertheless, during inflammation the host further limits metal availability by a mechanism named “nutritional immunity” (Behnsen et al., 2021[5]), a process that the host uses to limit metal concentrations, like zinc and iron, from pathogenic bacteria during infection (Hennigar and McClung, 2016[28]). This mechanism is thought to contribute to the limitation of disease severity and progression (Hennigar and McClung, 2016[28]). One of the proteins that the host uses for zinc sequestration is calprotectin (CP), an antimicrobial heterodimer of S100A8 and S100A9 (Skalny et al., 2021[86]). As this protein's expression is upregulated by IL-17 and IL-19, its levels are usually elevated in patients with inflammatory disorders and thus it is one of the many gut inflammation markers (Zeng et al., 2017[111]). However, some pathogenic bacteria like *Salmonella *overcome the CP-mediated Zn sequestration by expressing high affinity zinc binding proteins, like ZnuABC and ZupT permease (Kandias et al., 2009[37]; Skalny et al., 2021[86]). It is also interesting that some bacterial strains lacking the aforementioned zinc transporters express additional proteins like the siderophore yersiniabactin (Ybt), that besides iron can also bind zinc (Behnsen et al., 2021[5]).

## Zinc and Severe Acute Respiratory Syndrome Coronavirus 2 (SARS-CoV-2)

The COVID-19 pandemic, caused by SARS-CoV-2, counts more than 690 million cases and has led to nearly 7 million deaths worldwide (Worldometers.info, 2023[104]). SARS-CoV-2, that is a member of human-infecting CoVs (HCoVs) like the Middle East respiratory syndrome coronavirus (MERS-CoV) (Santacroce et al., 2021[84]), occupies the lower respiratory system and mostly results in viral pneumonia, but it can also lead to serious complications on vital organs with long-lasting effects (Forsythe and Barbar, 2021[21]). Numerous patients with COVID-19 infection develop severe pneumonia requiring hospitalization, or even admission to an intensive care unit (ICU). It is not uncommon for ICU patients to develop acute respiratory distress syndrome and multiple organ failures, as a result of excessive release of inflammatory cytokines, activation of procoagulating factors, and increased oxidative stress (Santacroce et al., 2021[84]). In such cases, artificial nutrition containing micronutrients like zinc is often necessary for malnutrition prevention (Santacroce et al., 2021[84]). It is worth mentioning that critically ill patients may demonstrate elevated use of zinc, and thus also present low serum zinc that is associated with a prolonged hospital stay (Jothimani et al., 2020[34]), a greater incidence of complications (Razzaque, 2021[75]) and a higher mortality rate (Santacroce et al., 2021[84]). Some studies have also found that patients with severe COVID-19 induced acute respiratory distress syndrome had some form of zinc deficiency and that low serum zinc is regarded as risk factor for serious disease development (Yasui et al., 2020[109]). The human angiotensin-converting enzyme 2 (ACE2) receptor is the key entry point for the SARS-CoV-2. Zn^2+^ binds to specific catalytic site in ACE2 modulating its activity but could have also the ability to coordinate Zn^2+^ ions in the same region where it binds the spike protein with a crucial impact on the recognition and interaction mechanism of ACE2-Spike (Pelucelli et al., 2023[69]). Additionally, zinc is essential for the proper development and function of immune system cells and its immunomodulatory effects could help the management of COVID-19 (Dhawan et al., 2022[18]).

## Zinc, Alzheimer’s Disease and Aging

Neurodegenerative diseases (NDs) are disorders which progress with time. Their common features are neuronal death in some areas of the brain, synaptic damage, and accumulation of protein aggregates, while the symptoms of NDs are memory and cognitive impairments that eventually lead to death. Dementia is a type of ND characterized by a progressive cognitive decline and decline of memory. It typically appears above the age of 65; it is estimated that Dementia now affects more than 50 million people worldwide (Gomes et al., 2020[24]). Alzheimer's disease (AD) is a common form of dementia, accounting for 70 % of its cases. The number of AD patients is expected to reach ~100 millions in 2050 (Liu et al., 2019[50]). The first symptom of the disease is amnesia which is called mild cognitive impairment (MCI) (Swerdlow, 2007[93]), while the subsequent stages of the disease (mild and moderate AD) show progressive cognitive and movement impairment. The brain of the affected people is characterized by atrophy of the cerebral cortex and hippocampus, narrowed gyri, widened sulci and expanded cerebrospinal fluid-filled ventricles, all of which contribute to language problems and affected information processing (Yamasaki et al., 2012[107]).

**Figure 2:**
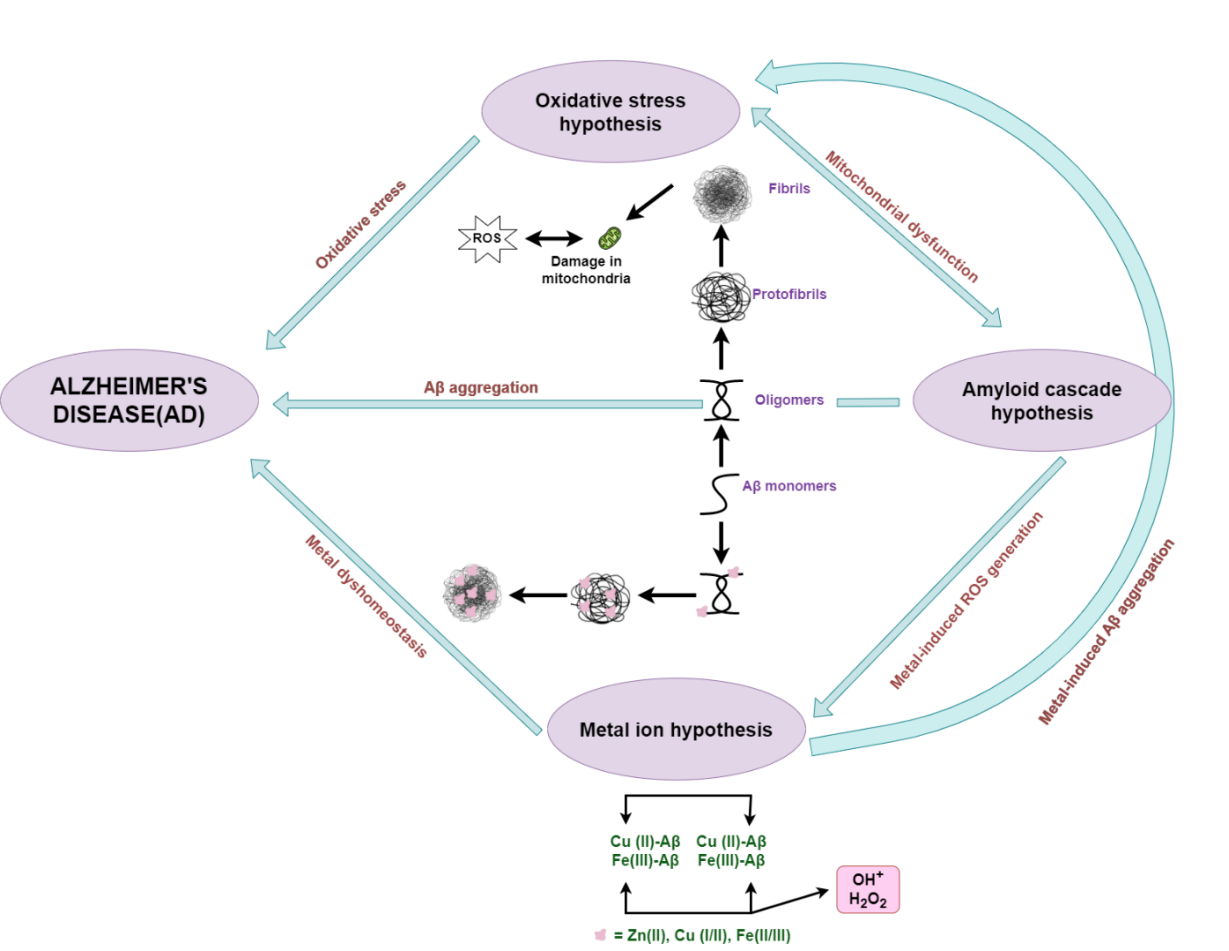
A simplified sketch that illustrates oxidative stress, metal ion and amyloid cascade mechanistic hypotheses as serious risk factors in AD. Metal ions promote ROS generation and, as a result, activate mitochondrial dysfunction and accumulation of ROS. Metal ions induce Aβ aggregation by binding to Aβ proteins. Aβ can also exhibit oligomeric transformation to fibrils, activating the mitochondria and generating more ROS. ROS cause damages to biomolecules and proteins in the brain and the AD risk increases. This picture has been adapted from the reference Fasae et al. (2021).

The pathobiology and biological chemistry of AD have been studied for almost four decades, with often controversial results. There are two pathological transformations in AD (Ma et al., 2020[53]). The first is intercellular deposition of amyloid plaques, which are formed by amyloid-beta (Aβ, vide infra) in the cerebral cortex. The second is intracellular accumulation of neurofibrillary tangles (NFTs), which are assembled by hyperphosphorylated tau proteins. Based on these transformations and other experimental results, there are several hypotheses to explain the formation of senile plaques as well as disease mechanisms (Fasae et al., 2021[20]; Kepp, 2017[40]; Narayanan et al., 2020[65]), which are briefly outlined below. The amyloid beta cascade hypothesis proposes that AD is initiated by the aggregation and deposition of Aβ peptide aggregates in the brain. Aβ is a fragment of the amyloid precursor protein (APP), a transmembrane protein which exists in the neuronal as well as other issues. Proteolytic cleavage of APP by secretases generates different polypeptides. Misfolding and aggregation by the Aβ_1-40_ and Aβ_1-41_ fragments results in the formation of amyloid plaques, a distinct characteristic of AD. The microtubule-associated protein tau (MAPT) hypothesis involves the tau protein, which exists almost exclusively in the neurons of the central nervous system. Its central role is the stabilization of microtubules in the axon of neurons. If the isoforms (four to six) of tau are hyperphosphorylated, they detach from microtubules and self-assemble inside the cell to form NFTs. Consequently, microtubules destabilize proteins and disable intracellular transport. Finally, the stability of tau aggregates and NFTs can inhibit neuronal transmission leading to cell death. The cholinergic hypothesis is related to acetylcholine (ACh), which is the first neurotransmitter that was ever identified. ACh has an important role in the central and peripheral nervous system (e.g., learning and memory) due to its use by cholinergic neurons. ACh is synthesized in certain neurons by choline acetyltransferase (ChAT) from choline and acetyl coenzyme A (acetyl-CoA) and decomposed by acetylcholinesterase (AChE) into choline and acetate. In AD, cholinergic neurons exhibit decreased choline uptake and decreased ACh release. The reduction of ChAT in AD has been correlated with several plaques and disease symptoms. The oxidative stress hypothesis is related to the production of reactive oxygen species (ROS). The oxidative stress results from the imbalance between oxidants and antioxidants in biological systems. The brain can be attacked by free radicals because of high oxygen consumption, increased amounts of fatty acids that line its membranes, reduced antioxidant efficiency and hydrogen ion removal from cellular macromolecules. Oxidative stress in the brain increases with age, while intense research studies have shown that in AD patients, there is increased oxidative stress, which leads to aggregation of Aβ peptides and NFTs. The metal ion hypothesis has been developed because high levels of free metal ions are essential for synaptic transmission in the brain. A disruption in the homeostasis (also called dyshomeostasis) of the 'natural' calcium, copper, iron and zinc ion levels is a confirmed feature of AD. These metal ions play significant roles in neuron signaling, inflammation, apoptosis, control of oxidative stress and cell proliferation. Generally, small variations in metal ion concentrations can shift their effects from beneficial to toxic within cells. Careful analysis in post-mortem brain tissues of AD patients has revealed elevated concentrations of Fe, Cu and Zn ions, while there is also strong evidence supporting the claim that coordination of β-amyloid with these ions enhances the aggregation of Aβ, triggering neuronal damage. An overview of several hypotheses as risk factors in AD is illustrated in Figure 2(Fig. 2) (Reference in Figure 2: Fasae et al., 2021[20]).

In the last three decades, there has been increasing evidence that biometal ions are associated with a variety of neurological disorders including AD. The possible role of Zn^2+^ in the pathology of AD was first reported in 1981 (Burnet, 1981[10]). The Zn^2+^ concentration in the brain is approximately 150 μM, 10 times higher than its concentration in the serum. This metal ion has a crucial role in the physiology and physiopathology of brain functions (Liu et al., 2019[50]; Portbury and Adlard, 2017[71]). Only ~15 % of brain Zn^2+^ is 'free' or chelatable, i.e., in a loosely bound or 'mobile' form (mZn) and can be located in various areas of the brain, including the cortex, the amygdala, the forebrain and the hippocampus; with the latter area being closely related to memory and learning. In this labile Zn^2+^ pool in the brain, complexes of this metal ion with proteins can be easily disrupted, making Zn^2+^ more labile. In the static Zn^2+^ pool (~85 %), the metal ion forms stable complexes with protein families, the most important of which are metallothioneins (MTs). There is a hypothesis that mZn may act as a neuromodulator (Radford and Lippard, 2013[74]); this hypothesis arises from the high concentration of mZn (>90 μΜ) within glutamatergic versicles, combined with the action of glutamate as a neurotransmitter. At the cellular level, mZn is loaded into presynaptic vesicles by the Zn^2+^ transport protein ZnT3, which modulates plasticity of glutamatergic neurons.

The most important regulators of Zn^2+^ homeostasis in the human brain are the Zn-Importing Proteins (ZIPs), the Zn Transporters (ZnTs) such as the ZnT3, and the MTs which act as buffering proteins that bind cytosolic Zn^2+^. The ZIPs and ZnTs behave in opposite manners. The former increases the levels of intracellular Zn^2+^ by directing this metal ion into the neurons and glial cells from either the vesicles or the extracellular environment. The latter decreases cytosolic Zn^2+^ levels by increasing vesicular uptake or efflux of Zn^2+^. The MTs have a double role because of their excellent affinity for binding Zn^2+^: First, they regulate the homeostasis of Zn^2+^, and, second, they have a protective role against oxidative stress; the latter arises from the fact that redox activity influences the high affinity and specificity of the coordination sites on metallothioneins (Fasae et al., 2021[20]). As far as their second role is concerned, upon oxidative stress the bound metal ion is released from Zn^2+^-binding proteins and results in the oxidation of the MTs and the formation of a disulfide MT-S-S-MT bond. Under reduced conditions, the disulfide bond is reduced to MT. This so-called 'Zn-metallothionein cycle' justifies the Zn^2+^-MT binding in the brain (Fasae et al., 2021[20]). When the brain environment favors the formation of ROS, structural rearrangement of MTs leads to Zn^2+^ release, and the concentration of the free metal ion increases. In contrast, in reduced environments, the MTs are reduced, and their thiol groups can sequester free Zn^2+^.

Both the excess and deficiency of Zn^2+^ have been associated with neurological problems since the 1980s (Itoh et al., 1983[31]). There is a huge literature addressing the problem if deficiency or excess of Zn^2+^ is related to AD. Detailed studies have provided strong evidence that serum levels of Zn^2+^ are lower in AD patients compared to those levels in the control group of patients (Brewer et al., 2010[8]). The idea is that Zn^2+^ contributes to AD pathology by promoting the accumulation of Aβ. A specific Zn^2+^-binding site is present in the cysteine-rich region of APP. This site is homologous in all the members of the APP family and amyloid precursor-like protein 1 and 2. The conclusion of these discoveries is that Zn^2+^ has a conserve role in the metabolic pathway and function of APP. The hyperphosphorylation of tau (vide supra) is a main feature of AD and Zn^2+^ plays a role in this phenomenon. It seems that Zn^2+^ mediates/assists the phosphorylation of the serine residue at position 214 of the tau protein, which leads to activation of the extracellular signal regulated kinases (ERK) pathway finally resulting in a decrease in microtubule stability (Kim et al., 2011[42]).

In summary, Zn^2+^ is neuroprotective at physiological concentrations (Fasae et al., 2021[20]; Liu et al., 2019[50]; Narayanan et al., 2020[65]; Portbury and Adlard, 2017[71]), but neurotoxic at high concentrations. Zn^2+^ overload causes acceleration of Aβ aggregation, alters processing of APP, induces tau toxicity by binding to it and inducing its hyperphosphorylation, promotes tau aggregation and enhances oxidative stress. It is involved in the pathogenesis of AD by various biochemical mechanisms such as Aβ oligomerization, tau hyperphosphorylation and initiation of the ERK pathway, all of which leading to accumulation of NFTs. 

There is now strong evidence that biometal ions (Fe, Cu, Zn) play a fundamental role in modulating Aβ function and are related to the pathogenesis of AD (Fasae et al., 2021[20]; Kepp, 2017[40]). This experimental evidence has led to several ideas for therapeutic approaches to AD. Three main approaches have been developed (Kepp, 2017[40]). The first is to target metal ion dyshomeostasis per se, without taking into account the Aβ. A second is to target the toxic metal ion-Aβ species either by preventing their binding or their combined function. The third strategy is to address the downstream toxicity caused, mainly by the employment of antioxidant or anti-inflammatory drugs.

**Figure 3:**
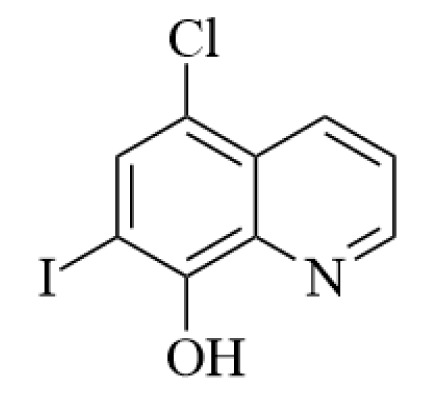
The structural formula of 5-chloro-7-iodo-quinolin-8-ol (CQ) which played a significant role in the development of drugs for AD.

The *first strategy* involves the use of chelating ligands with a specificity for Zn^2+^; assuming that the levels of this metal ion are related with pathogenic imbalances, the ligands (i.e., the drugs) are capable of reconstituting normal Zn^2+^ levels within the neuron. Many chelators reduce amyloid plaque formation, down-regulate APP expression and decrease the Aβ levels. A typical drug associated with this approach is 5-chloro-7-iodo-quinolin-8-ol or clioquinol (CQ) (Figure 3(Fig. 3)). However, this ligand also binds strongly to Cu^2+^ and likely interferes with the homeostasis of this redox-active metal; unfortunately, CQ induces subacute myelo-optic neuropathy (SMON) and was withdrawn from the market in the 1980s. Today intense research is focused on the design of Cu^2+^/Zn^2+^ selective chelating agents that can rebalance the intra-neuronal Cu^2+^/Zn^2+^, which probably is involved in the disease (Kepp, 2016[41]).

The central goal of the *second strategy* is to target the direct interaction between metal ions (including Zn^2+^) and Aβ in order to alter the properties of the complexes. CQ is a chelating ligand that interacts with metal-Aβ complexes and it is thus an example of a metal-protein attenuating compound (Kepp, 2017[40]). There are two mechanistic schemes that have been proposed. In the first, the ligand is coordinated to the free metal ion decreasing the concentration of metal-Aβ complex. In the second mechanistic proposal, the ligand is coordinated to the metal ion and Aβ simultaneously forming a ternary complex which modifies the behavior of the metal-Aβ complex. The clarification of the mechanism will help scientists to develop future drugs. Small molecules, such as synthetic flavonoids and small peptides, that can probe and interact with metal-Aβ complexes are intensely studied, and they exhibit fibrillation and neurotoxicity.

The *third strategy *involved, oxidative stress in AD, as it is considered as the age trigger of the disease (Kepp, 2017[40]). Thus the role of antioxidants on AD seems beneficial, although there are various opinions in the scientific community (Luchsinger et al., 2003[52]). Molecules pursued as AD treatment schemes include ascorbic acid, α-tocopherol, polyphenols, phenylpropanoids and curcumin derivatives. The latter three are considered as multifunctional natural compounds against AD because they combine more than one function. For example, polyphenols are antioxidants, but they can also reduce Aβ aggregation via direct interaction with Aβ, coordination with Zn^2+^ (and other biometal ions) or as ternary complexes. Inflammation is also a feature of AD. Thus, it would probably be beneficial to reduce inflammatory responses in the context of treatment strategies. Two families of drugs are used; steroid-hormone-based and non-steroid anti-inflammatory drugs (NSAIDs) (McGeer et al., 2006[60]). Meta-analyses have confirmed the beneficial effect for many of them (Wang et al., 2015[99]). Experiments have shown that the NSAIDs ibuprofen and sulindac decrease the levels of longer Aβ forms (mainly Aβ_1-42_), with corresponding increase in shorter isoforms (Weggen et al., 2001[102]). NSAIDs have proven to increase Zn^2+^ concentrations in the brain, which in turn regulate cleavage of the APP-related proteins (Kepp, 2017[40]).

In summary, AD is a multiparameter complex disease outside the concept 'one disease, one target protein, one drug' (Robert et al., 2015[81]). Today there is strong evidence linking AD to (i) Aβ-aggregation, (ii) tau pathology, and (iii) metal ion dysregulation. These targets are probably interconnected. It has been proven that there is loss of copper, iron and zinc ions homeostasis in AD; post-mortem analysis of amyloid plaques reveals an excessive accumulation of these ions in the brains of AD patients. The restoration of metal ion homeostasis is a valuable challenge for AD chemotherapeutic schemes. More attention should be paid to the necessity of developing specific chelating agents in order to limit toxic side effects (Robert et al., 2015[81]). Success towards this direction has recently been achieved (Liu et al., 2019[50]). As far as Zn^2+^ is concerned, chelation has been shown to limit the formation of senile plaques (the main hallmark of AD). The modulation of the levels of this metal ion by non-toxic chelators may provide scientists with a potential therapeutic strategy for AD (Robert et al., 2015[81]).

However, zinc is also connected to the aging process as a whole. Zn homeostasis is disturbed as part of the aging process, in part due to dietary deficiencies that are especially frequent in the elderly, resulting in lower amounts of the ion and elevated MT levels that lead to zinc sequestration. As a result, this low zinc bioavailability contributes to the loss of immunological responses, consisting an important risk factor for infection relapses in the elderly (Cabrera, 2015[11]). Aging is usually followed by a severe decrease in the production of IL-2 by the immune cells, making seniors more vulnerable to a number of age-related disorders and interestingly, a typical cause of decreased cytokine production is zinc deficiency. Nonetheless, the molecular mechanisms underlying this specific phenomenon have not been elucidated yet. Finally, it has been proven that the levels of zinc in synaptic vesicles along with the expression of ZnT3, the transporter in charge of the metal's packaging, decrease with advanced age and result in age-dependent deficits in learning and memory ability (McCord and Aizenman, 2014[59]; Niu et al., 2020[66]).

## Zinc Supplementation

There is evidence that zinc supplementation could be beneficial for cancer patients as a part of treatment, either alone or in combination with other drugs. The elevation of zinc levels into cancer cells has given various results. While a small dosage of zinc may not suffice for a biological result, but at the same time high doses of zinc could result to toxicity (To et al., 2020[96]). For example, in the case of prostate cancer patients heavy zinc supplementation could further increase the risk for cancer development due to immunosuppression (Zhang et al., 2022[114]). Zinc supplementation has had a positive effect in dysgeusia, dermatitis and radiotherapy- induced inflammation of oral and oropharyngeal mucosa that are common side effects in cancer patients (Hoppe et al., 2021[29]). More specifically, there is evidence in favor of zinc carnosine (ZnC) supplementation use for the treatment of mucosal damage and epithelial tissue, side effects that accompany radiation therapy (Tang et al., 2022[95]).

Zinc supplementation and zinc's antiviral properties have been extensively studied in the cases of hepatitis C virus (HCV), human immunodeficiency virus (HIV) and other coronaviruses (Jothimani et al., 2020[34]). The main idea is that zinc probably inhibits RNA synthesis and topoisomerase activity, alters the proteolytic processing of viral polyproteins, and influences viral uncoating, binding and replication thus limiting viral load (Chasapis, 2018[12]; Skalny et al., 2020[87]). Zinc may also enhance membrane integrity and block the entry of the virus in the cell (Kumar et al., 2020[44]). Finally, zinc supplementation can act synergistically with antiviral therapy administration, as demonstrated in HCV, HIV and SARS-CoV-1 cases (Kumar et al., 2020[44]). To conclude, there is compiling evidence suggesting that zinc supplementation is in general beneficial for the management of COVID-19.

## Conclusion

Zinc has been studied for its potential benefits in several diseases and conditions. In this review its role in particular medical conditions including cancer, COVID-19, aging neurological disorders such as Alzheimer's disease, and its action for aging and the gut microbiome was discussed. The studies on zinc and cancer are still in the preliminary stages and more research is needed to determine the efficacy and correct dosage of zinc in the treatment of cancer. Currently, there are some studies suggesting a possible role of zinc in the prevention and treatment of COVID-19. However, it is important to note that the evidence is limited and there is still no clear scientific conclusion regarding the effectiveness of zinc in fighting disease. Some studies have suggested that zinc supplementation in the context of COVID-19 might reduce symptom severity and speed recovery, but more research is needed to confirm those findings. Researchers have been actively investigating the role of zinc in the development, progression, and treatment of Alzheimer's disease. Some research has suggested that zinc metabolism may be impaired in people with Alzheimer's disease, leading to abnormal zinc levels in the brain. Under normal conditions, zinc plays an important role in brain functioning, including the formation and communication of neurons. However, excessive amounts of zinc in the brain could contribute to the formation of beta-amyloid protein plaques characteristic of Alzheimer's disease. Some *in vitro* and animal model studies have suggested that zinc may play a role in amyloid-beta protein toxicity and disease progression. However, studies in humans have produced conflicting results and the evidence is still limited. Some studies have shown that a zinc deficiency can alter the composition of the GM, favoring the growth of certain pathogenic or opportunistic bacteria at the expense of beneficial bacteria. Zinc plays a fundamental role in the health of the intestinal microbiome, influencing the integrity of the intestinal barrier, the functionality of the intestinal immune system and the composition of the microbiome itself. Ensuring an adequate supply of zinc through a balanced diet or, if necessary, through supplements, can help maintain a healthy gut microbiome and is conducive to overall health. 

In conclusion, it is important to note that the therapeutic use of zinc for these diseases is not yet well established and further research is needed to confirm its efficacy and determine the appropriate dosage. Additionally, taking zinc supplements or increasing dietary zinc intake should always be supervised by a physician, as excessive zinc dosages can be harmful resulting to toxicity.

## Declaration

### Acknowledgments

C.T.C. and D.P.K. would like to thank the National Research Foundation (NHRF) for supporting the research work by providing a Research Seed Grant.

### Conflict of interest

The authors declare no conflict of interest.

## Figures and Tables

**Table 1:**
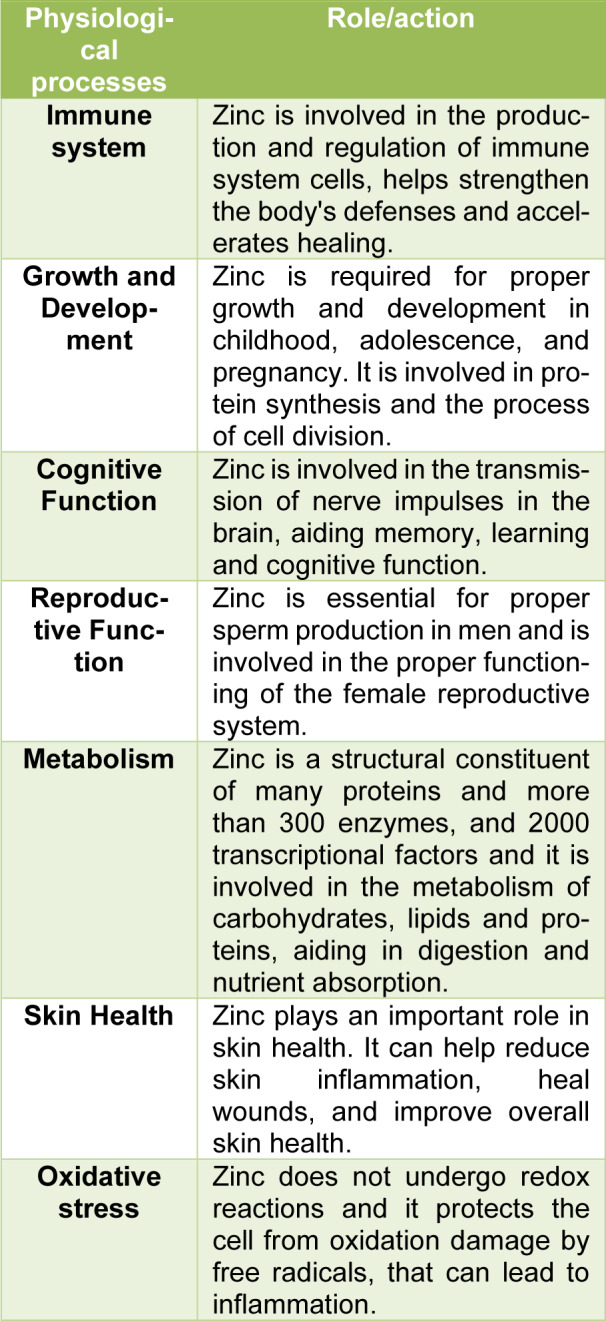
Role of zinc in human body
